# Intermittent Screening and Treatment versus Intermittent Preventive Treatment of Malaria in Pregnancy: A Randomised Controlled Non-Inferiority Trial

**DOI:** 10.1371/journal.pone.0014425

**Published:** 2010-12-28

**Authors:** Harry Tagbor, Jane Bruce, Mitchell Agbo, Brian Greenwood, Daniel Chandramohan

**Affiliations:** 1 Department of Community Health, School of Medical Sciences, Kwame Nkrumah University of Science and Technology, Kumasi, Ghana; 2 Juaben Government Hospital, Juaben, Ashanti, Ghana; 3 Department of Infectious and Tropical Diseases, London School of Hygiene & Tropical Medicine, London, United Kingdom; Kenya Medical Research Institute, Kenya

## Abstract

**Background:**

The effectiveness of intermittent preventive treatment of malaria in pregnancy (IPTp) may be compromised by the spread of resistance to sulphadoxine/pyrimethamine (SP) across Africa. But little informtion exists on alternative drugs for IPTp or alternative strategies for the prevention of malaria in pregnancy. Therefore, we have investigated whether screening with a rapid diagnostic test and treatment of those who are positive (IST) at routine antenatal clinic attendances is as effective and as safe as SP-IPTp in pregnant women.

**Methods and Findings:**

During antenatal clinic sessions in six health facilities in Ghana held between March 2007 and September 2007, 3333 pregnant women who satisfied inclusion criteria were randomised into three intervention arms (1) standard SP-IPTp, (2) IST and treatment with SP or (3) IST and treatment with amodiaquine+artesunate (AQ+AS). All women received a long-lasting insecticide treated net. Study women had a maximum of three scheduled follow-up visits following enrolment. Haemoglobin concentration and peripheral parasitaemia were assessed between 36 and 40 weeks of gestation. Birth weight was measured at delivery or within 72 hours for babies delivered at home. Parasite prevalence at enrolment in primigravidae and in multigravidae was 29.6% and 10.2% respectively. At 36–40 weeks of gestation the prevalence of asymptomatic parasitaemia was 12.1% in study women overall and was very similar in all treatment groups. The risk of third trimester severe anaemia or low birth weight did not differ significantly between the three treatment groups regardless of gravidity. IST with AQ+AS or SP was not inferior to SP-IPTp in reducing the risk of low birth weight (RD = -1.17[95%CI; -4.39-1.02] for IST-SP vs. SP-IPTp and RD = 0.78[95%CI; -2.11-3.68] for IST-AQAS vs. SP-IPTp); third trimester severe anaemia (RD = 0.29[95%CI; -0.69-1.30] for IST-SP vs. SP-IPTp and RD = -0.36[95%CI;-1.12-0.44] for IST-AQAS vs. SP-IPTp).

**Conclusion:**

The results of this study suggest that in an area of moderately high malaria transmission, IST with SP or AS+AQ may be a safe and effective strategy for the control of malaria in pregnancy. However, it is important that these encouraging findings are confirmed in other geographical areas and that the impact of IST on placental malaria is investigated.

**Trial Registration:**

ClinicalTrials.gov NCT00432367 [NCT00432367]

## Introduction

*Plasmodium falciparum* infection in pregnancy causes maternal anaemia and low birth weight associated with parasitisation of the placenta [1]. The World Health Organization recommends intermittent preventive treatment (IPTp) with sulphadoxine-pyrimethamine (SP), insecticide-treated bed nets (ITNs), and effective case management as measures to protect against these outcomes [2].

Intermittent preventive treatment linked to antenatal care will remain an effective and sustainable strategy for the prevention of malaria in pregnancy provided that the antimalarial drug used is efficacious, safe, tolerable, cheap, and easy to administer, preferably as a single dose [3]. So far SP is the only drug which has these attributes. Thus, as resistance to SP increases and spreads across Africa, [4], [5], [6] the effectiveness of the IPT strategy may be compromised. Trials are under way to evaluate alternative drugs that could be used for IPTp in place of SP but none of the potential candidates have the favourable characteristics of SP when used in areas where parasites are sensitive to this drug.

In the absence of an effective drug to replace SP for IPTp alternative strategies for the prevention of malaria in pregnancy need to be evaluated. Insecticide treated bed nets (ITNs) used during pregnancy are beneficial to both mother and her newborn baby [7]and so strenuous efforts are being made to increase ITNs accessibility to pregnant women across Africa [8]. It is not clear whether women who are protected by an ITN also need IPTp. In a study conducted in western Kenya, the combination of IPTp and ITNs was slightly more effective than ITNs alone (PE 56% vs. 42%) but only in primigravidae. [9]. In the Gambia, where the prevalence of HIV infection and SP resistance are low, no beneficial effect from SP-IPTp on anaemia or birth weight was seen in multigravidae, with the exception of a small sub-group of women who did not use a bed net [10].

Screening for malaria infection using a malaria rapid diagnostic test (RDT) at scheduled antenatal clinic visits and treatment of women who are positive with an effective antimalarial drug (IST), combined with effective vector control, provides a potential, alternative strategy to SP-IPTp. The WHO's current recommendation of four scheduled antenatal clinic visits, one at booking and three subsequent visits 4 to 8 weeks apart, provides a potential framework for implementation of an IST strategy. This strategy may be considered in parts of Africa where resistance to SP is increasing at alarming rates and in situations of low exposure to malaria as in The Gambia and Zanzibar where the incidence of malaria has gone down [11], [12], [13] or in Asia where transmission is generally low [14].

Intermittent screening and treatment combined with vector control may be an especially attractive option in such communities where continuing use of IPTp with SP will result in a large proportion of pregnant women receiving SP unnecessarily. This is also the case for communities where the incidence of malaria is highly seasonal. In these areas, many women receive IPTp during months of the year when the risk of malaria is minimal.

Whether a screening and treatment strategy would prove to be as effective as SP-IPTp is not known. Therefore, we have undertaken a randomised, controlled trial in an area of Ghana with moderate malaria transmission to determine whether IST using SP or amodiaquine+artesunate (AQ+AS) is as effective in preventing maternal anaemia and low birth weight as SP-IPTp. The costs and acceptability to women and providers of such a strategy have also been assessed.

## Methods

### Overall study design

The protocol for this trial and supporting CONSORT checklist are available as supporting information; see Checklist S1 and Protocol S1. It was an individually randomised, open, controlled trial was undertaken to investigate whether screening for malaria with an RDT and treatment of women with a positive test with either SP or AS+AQ was not inferior to SP-IPTp in the prevention of anaemia in pregnancy or low birth weight.

### Study population

The study was conducted in the Ejisu-Juaben and Afigya-Sekyere East districts of the Ashanti Region of Ghana from March 2007 to September 2008. Malaria transmission in this area is perennial but with a peak in the rainy season. The predominant parasite is *P. falciparum*. The entomological inoculation rate in the neighbouring area of Kintampo is about 250 infectious bites per year. HIV prevalence in Ashanti region is reported to be 3.0% and 2.2% in the general population and in pregnant women respectively (Ghana Health Service 2007 Annual Report).

**Figure 1:**
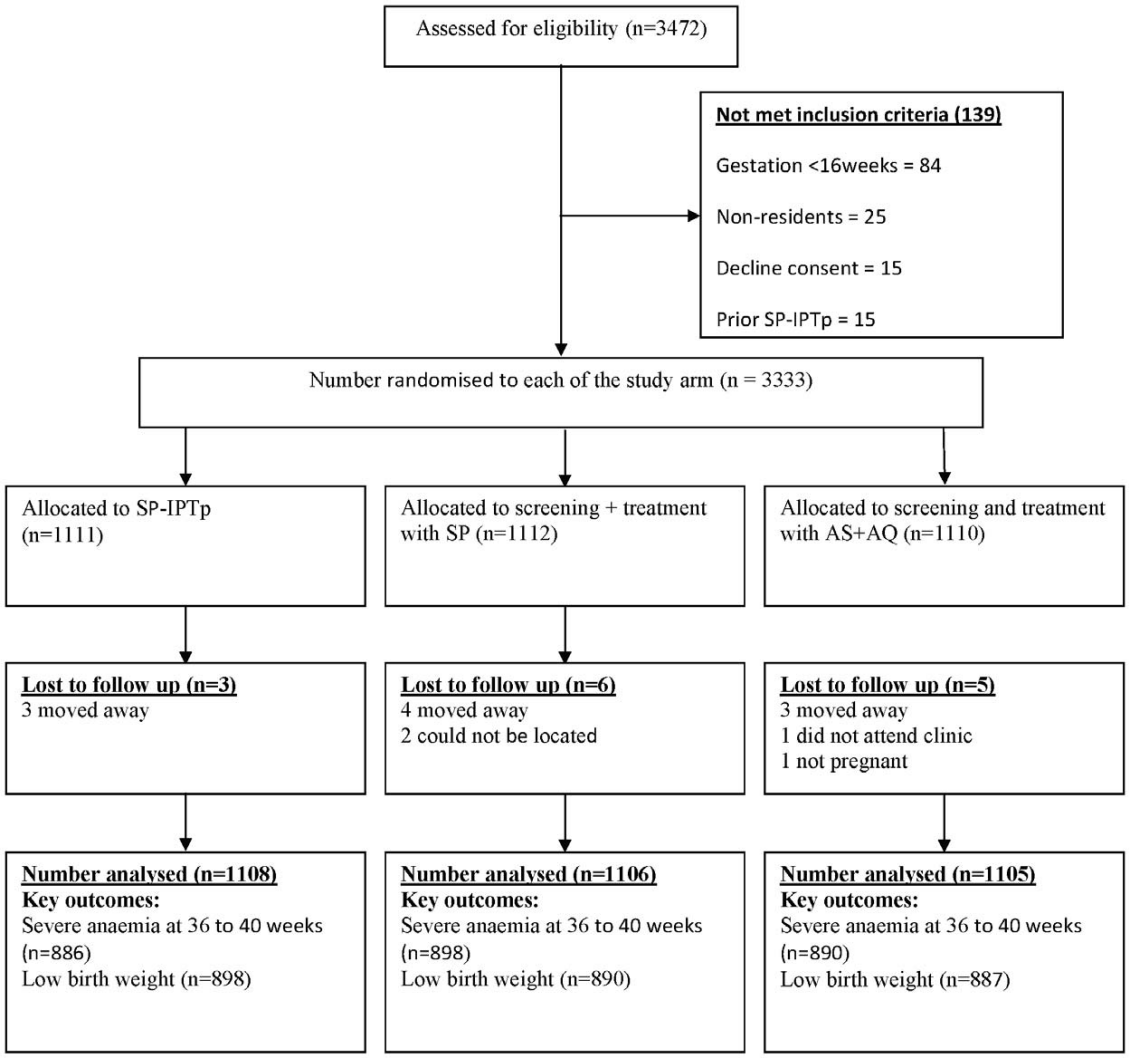
CONSORT chart showing enrolment and follow-up status of women in each study arm.

Enrolment was undertaken at the antenatal clinic of three district hospitals and three health centres in the study area. The study population comprised pregnant women of all parities who presented at the antenatal clinics with a gestational age of 16 to 24 weeks at their first booking. Women who were temporary residents, had had a prior dose of SP-IPTp, had a haemoglobin concentration <5 g/dl, gave a history of sensitivity to SP, amodiaquine or an artemisinin, had an illness requiring hospital admission or declined to join the trial were excluded (Figure 1).

### Ethics

The study protocol was approved by the ethics committee of the London School of Hygiene and Tropical Medicine and the Committee on Human Research and Publications Ethics of the School of Medical Sciences, Kumasi – Ghana. A Data, Safety and Monitoring Board (DSMB) approved the protocol, standard operating procedures and analysis plan.

### Sample size

Sample size was calculated on the assumption that the prevalence of moderately severe anaemia (Hb<8 g/dl) in the third trimester of pregnancy and of low birth weight in women in the SP-IPTp arm of the study would be at least 12% and 6% respectively, figures based on findings from a study undertaken in The Gambia [15]. To establish that IST was not inferior to SP-IPTp we decided that it was necessary to show that the differences in the percentages of women with moderately severe third trimester anaemia or low birth weight between the IST groups and the SP-IPTp group should not be more than 5% or 4% respectively, differences that would be clinically important. To meet these criteria with 90% statistical power and allowing for 20% loss to follow-up it was calculated that 1,110 women were needed in each study arm to give a total sample size of 3,330.

### Procedures

After written informed consent had been obtained, eligible women of all parities were randomised to one of the three treatment groups described above. Women who declined to participate were given SP-IPTp according to the national guidelines. The following process was followed to randomise women into the study arms. A list of random numbers was computer generated as identification numbers, randomly allocated to treatment groups and grouped in blocks of 15 by an IT specialist who did not participate further in the study. The list of identification numbers with their corresponding treatment groups was printed and cut into slips. Fifteen slips each with an identification number and an allocated treatment group were sealed in an envelope. During enrolment an eligible pregnant woman was asked by the recruitment team to pick a slip from the sealed envelope. The allocated treatment group printed on each slip indicated which treatment arm the women belonged to and the corresponding identification number was used to identify her. This constituted entering the study, and treatment group allocation was binding on the recruiting team and the woman from this point. Another envelope was opened only when the contents of the previous one had been exhausted. The PI and all other project staff were blinded to the randomisation process and treatment allocation.

At enrolment, a finger prick blood sample was obtained for measurement of haemoglobin concentration, preparation of thin and thick blood films for malaria parasite counts and preparation of a filter paper blood spot. During the last 3 months of the trial, women in treatment group 1 who had a positive blood film and received SP-IPTp were seen again on day 14 and day 28 after treatment and a further blood film and blood spot obtained to check on the response to treatment with SP. HIV screening was offered to all pregnant women study women as part of the routine antenatal services recommended in Ghana with an option for treatment but the results of HIV screening were not available to the investigators. Women in the SP-IPTp arm received an initial dose of SP (1500 mg sulphadoxine/75 mg pyrimethamine) as a single dose. Pregnant women in treatment groups 2 and 3 were screened for malaria infection with an OptiMAL® dipstick, a lactate dehydrogenase (LDH) based RDT. The OptiMAL® dipsticks were purchased from DiaMed AG, Cressier, Switzerland who supplied and organised transportation to the study site in batches as and when needed,. The test kits were kept at room temperature always at the study site. The viability of the kits were tested monthly throughout the study period using positive test controls obtained from the manufacturer. The tests were performed and interpreted by the study team following the manufacturer's instructions. Women in treatment group 2 were treated with a single dose of SP (1500 mg sulphadoxine/75 mg pyrimethamine) if the RDT was positive. Women in treatment group 3 were treated with AQ+AS (AQ-300 mg + AS-100 mg twice daily for 3 days) if the RDT was positive. All treatments with SP and the first dose of AQ+AS treatment were administered by a member of the study team but doses 2 and 3 of AQ+AS were given to study women to take at home unsupervised. Women in groups 2 and 3 received no antimalarial treatment or IPT if their RDT results were negative. All women received a daily supplement of ferrous sulphate (200 mg) and folic acid (4 mg) tablets as part of the routine antenatal care services provided in Ghana throughout the study period. SP was purchased from Kinapharma Ltd, Ghana and AQ+AS was provided by the Ministry of Health, Ghana. All study women received a long-lasting insecticide treated bed net (LLIN) and instructions on how to use this at enrolment.

Study women were asked to attend for follow-up antenatal care and IPTp or screening at 24, 32 and 36 weeks of gestation. At the 24 and 32 week visits, women in treatment group 1 received SP-IPTp whilst women in treatment groups 2 and 3 were screened with the RDT and, if positive, treated with SP or AS+AQ according to the allocation arm. As part of safety assessment, all women were visited at home by a trained community health worker to record any complaints that the women might have seven days after each scheduled antenatal visit.

Blood samples were obtained from all study women between 36 and 40 weeks of gestation (before delivery) for determination of haemoglobin concentration and for preparation of thin and thick blood films. However, the smears were read retrospectively and so the results were not available for the point of care. Any study woman who presented with a history of fever or other features suggestive of malaria between scheduled antenatal visits was screened for malaria using an RDT and treated with quinine (30 mg/kg in divided doses daily for 5 days) regardless of treatment group.

Information on the outcome of pregnancy was obtained for 2706 of the 3333 study women (81.2%), 2144 of whom delivered at a health centre or hospital and 562 at home. If a woman delivered at a health facility, birth weight was recorded by a midwife who was unaware of the treatment group of the woman who she was attending. Women who delivered at home were traced through a network of trained community health workers within 72 hours of delivery and the infant's weight was measured at home. The occurrence of miscarriages, still births, neonatal deaths and the presence of congenital abnormalities was recorded by midwives. If any congenital abnormality was suspected, a full examination, including a neurological assessment, was undertaken by a qualified medical doctor. Field teams visited all women and babies at approximately 6 weeks post delivery to obtain reports of any neonatal adverse events and a blood film was obtained from mothers at this time. We could not determine the prevalence of placenta malaria and efficacy of SP in postpartum study women as proposed due respectively to inadequate funding and difficulty in finding postpartum women with parasitaemia.

An experienced microscopist, unaware of treatment group assignment, read all the blood films and quantified parasitaemia against 200 leucocytes in thick blood smears. A thick blood film was declared negative only after examination of 100 high power fields (HPF). An independent expert microscopist from the Noguchi Memorial Institute of Medical Research read ten percent of all blood slides obtained at enrolment and on follow up visits for quality assurance. Agreement between the study microscopist and the reference microscopist was 91.7% and a kappa of 0.83. Haemoglobin (Hb) concentration was measured using Hb 301 Hemocues (HemoCue AB, Angelholm, Sweden). Paired samples from women who failed treatment with SP were tested for molecular markers to differentiate reinfections from recrudescences using genetic markers as described previously [16]. HIV testing was not part of the study but it is likely that some HIV positive may have been included in the sample.

### Statistical methods

Stata version 10 (StataCorp, College Station, Texas) was used for data analyses. The primary objective of the study was to demonstrate that the risk of third trimester moderately severe anaemia (Hb<8 g/dl) in the IST groups was no more than 5% greater than in the SP-IPTp arm. Secondary objectives were to demonstrate that the risks of low birth weight (BW<2500 g), spontaneous abortions, intrauterine deaths/stillbirths, neonatal and maternal mortality were not more than 4% higher in women in the IST groups than in women who received SP-IPTp. The principal analysis of primary and secondary outcomes was per protocol but an "intention-to-treat" analysis was also undertaken according to a statistical analysis plan approved by the Data and Safety Monitoring Board. In the per protocol analysis, only data from women who remained within their randomization group, received two courses of SP-IPTp (Group 1) or were screened twice using an RDT at scheduled visits (groups 2 and 3) and in whom the primary outcome had been recorded were considered for analysis. In the intent-to-treat analysis, women were included if they had received an initial treatment of IPTp or had had an initial screening test done and provided that an outcome had been recorded.

The proportion of the per protocol and intention-to-treat populations experiencing each primary and secondary outcome for the treatment groups, and the associated 2-sided 95% CI for the difference, was estimated using the generalized linear model. To declare non-inferiority with a significance level of 0.05%, the upper boundary of the 2-sided 95% CI for the estimated treatment effect (risk difference) had to be below the pre-defined non-inferiority margins (Δ) of 5% and 4% for third trimester severe anaemia and low birth weight respectively. We controlled for gestational age at enrolment, gravidity, baseline parasitaemia and anaemia using binomial regression.

## Results

### Baseline demographic and clinical characteristics

A total of 3333 of the 3472 potentially eligible pregnant women who were screened (96%) were enrolled into one of the three treatment groups (Figure 1). At the end of follow up, 2674 (80.2%) evaluable records for third trimester haemoglobin concentration and 2675 (80.2%) records for birth weight were available.

Baseline characteristics of women in the three treatment groups were very similar at enrolment (Table 1). In all three treatment groups, more than half of the women were aged between 20 and 30 years with an overall mean age of 26.6 years. About ninety percent of the women had received a formal education, mainly up to secondary school level (70%). Fifty percent of the study population households already owned a bed net. Twenty-three percent of women were primigravidae and twenty-three percent were secundigravidae. About 4% of women had a haemoglobin concentration below 8 g/dl; the mean Hb overall was 10.97 g/dl. Anaemia at enrolment was associated significantly with asymptomatic malaria parasitaemia (p<0.05). The prevalence of malaria infection on presentation at the antenatal clinic in women in the SP-IST and AQAS-IST groups as determined by the OptiMAL® RDT was 22.9% (RDTs were not done in women in the SP-IPTp group). Asymptomatic parasitaemia as determined by microscopy was present in 16.3% of study women overall with parasite densities less than 1000/µL in 99% of them. *P. falciparum* was the most prevalent malaria species. *P. malariae* and *P.ovale* were also detected but only few women (<3%) carried these species. Additional blood samples were obtained 14 and 28 days after administration of a first dose of SPI-IPTp from 71 women who were parasitaemic on presentation. Eleven (15.5%; 95% CI 7.9%-26.0%) were positive by day 28. PCR corrected parasitological failure on day 28 was 5.6% (95% CI 1.6%-13.8%). The overall prevalence of HIV in pregnant women who agreed to screening in the study clinics during the period of the trial was approximately 1.5%.

**Table 1:** Comparison of demographic and baseline characteristics of study women.

	SP-IPTp		IST-SP		IST-AQAS		Total	
	(N = 1111)		(N = 1112)		(N = 1110)		(N = 3333)	
	n	(%)	n	(%)	n	(%)	n	(%)
**Age [years]**	Q							
<20	107	9.8	113	10.4	107	9.8	327	10.0
20–24	326	30.0	321	29.5	351	32.2	998	30.5
25–29	336	30.9	324	29.7	303	27.8	963	29.5
30+	318	29.3	332	30.5	330	30.3	980	30.0
Mean (SD)	26.5	5.9	26.6	6.0	26.5	6.0	26.6	6.0
Median (IQR)	26	8	26	8	26	8	26	8
**Educational level reached**								
None	72	9.1	90	11.2	87	10.7	249	10.3
Primary	145	18.3	132	16.4	135	16.6	412	17.1
Junior secondary school	489	61.7	511	63.3	485	59.6	1485	61.5
Senior secondary school	75	9.5	50	6.2	85	10.4	210	8.7
Tertiary	12	1.5	24	3.0	22	2.7	58	2.4
**Occupation**								
Farmer	95	12.1	106	13.2	104	12.9	305	12.7
Housewife	137	17.4	127	15.8	120	14.9	384	16.0
Salary Worker	356	45.2	352	43.8	375	46.5	375	45.2
Trader	29	3.7	38	4.7	39	4.8	39	4.4
Other	171	21.7	181	22.5	168	20.8	168	20.8
**Households with a bed net**								
No	477	47.9	464	45.8	507	50.1	1448	47.9
Yes	518	52.1	550	54.2	505	49.9	1573	52.1
**Sleep under net last night**								
No	221	27.6	241	30.0	235	29.6	697	29.1
Yes	580	72.4	563	70.0	558	70.4	1701	70.9
**Gravidity**								
3 or above	618	55.7	588	53.0	606	54.7	1812	54.5
2	246	22.2	276	24.9	244	22.0	766	23.0
1	245	22.1	246	22.2	258	23.1	749	22.5
**Haemoglobin (g/dl)**								
<8	35	3.2	38	3.4	47	4.2	120	3.6
8–10.9	564	50.7	597	53.7	595	53.6	1756	52.7
11 or above	512	46.1	477	42.9	468	42.2	1457	43.7
Mean (SD)	10.7	1.4	10.6	1.4	10.6	1.4	10.6	1.4
Median (IQR)	10.8	1.7	10.7	1.9	10.7	1.9	10.7	1.9
**Baseline Parasitaemia**								
No	926	83.5	925	83.3	937	84.5	2788	83.8
Yes	183	16.5	186	16.7	172	15.5	541	16.3
Geometric mean	198	10.2	197	10.8	187	10.7	582	10.5
parasite density								

### Study outcomes – per protocol analysis

**Figure 2:**
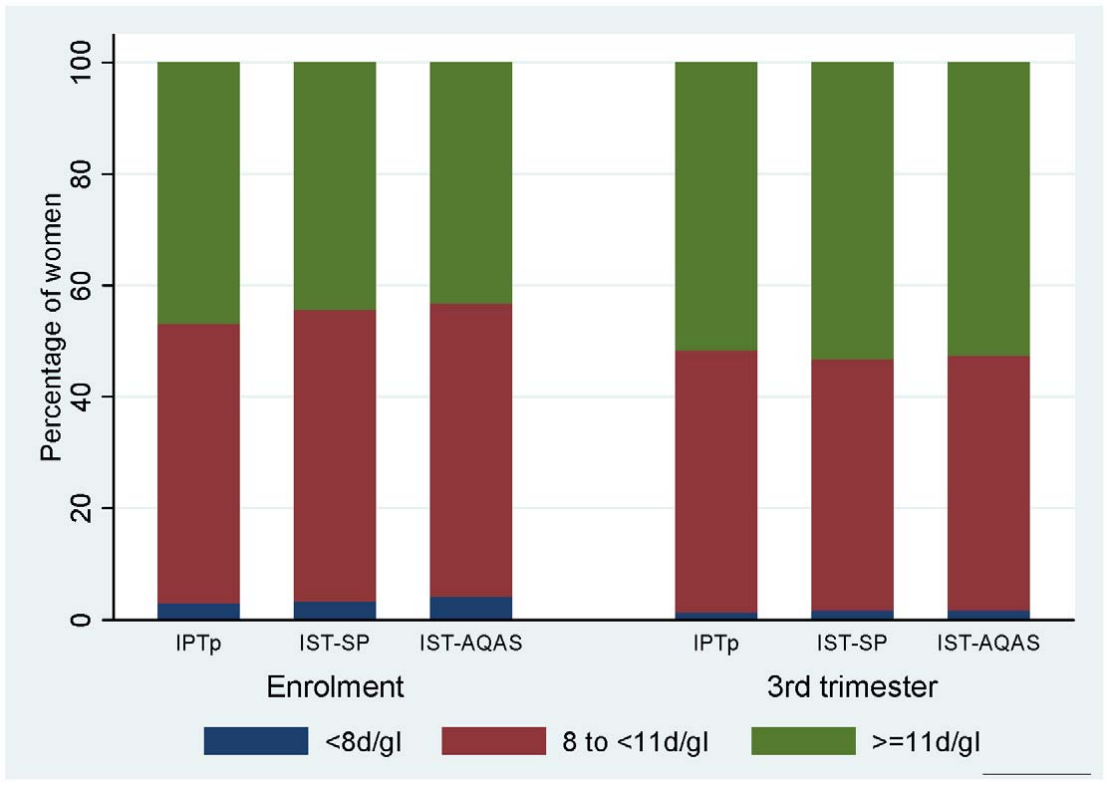
Mean change in Hb concentrations at 36 weeks gestation over baseline.

At 36–40 weeks of gestation (before delivery) the prevalence of severe anaemia (Hb<8 g/dl) and moderate (8< = Hb<11 g/dl) anaemia was respectively 1.7% and 45.9% overall and was similar in all treatment groups; similarly asymptomatic parasitaemia was 12.0% in study women overall and was very similar in all treatment groups. The mean Hb concentration of all women between 36 and 40 weeks of gestation was 11.0 g/dl and was similar in all the groups. Generally, there was a significant increase in mean Hb concentration of 0.29 g/dl at 36–40 weeks gestation over the baseline Hb concentration (Figure 2). The increase was higher in women with malaria parasitaemia at baseline (0.87 g/dl) compared to non-parasitic women (0.21 g/dl) but there was no significant difference in the increase in haemoglobin concentration during the course of pregnancy between the three treatment groups. The overall prevalence of low birth weight was 11.0% in the study women and did not differ significantly among the study groups (Table 2).

**Table 2:** Comparison^a^ of key outcomes in women enrolled in SP-IPTp or IST groups.

	SP-IPTp		IST-SP			IST-AQAS			Total	
	n	%	n	%	p-value^b^	n	%	p-value	n	%
**Haemoglobin** c										
**Hb<8 g/dl**	12	1.4	15	1.7	**0.65**	15	1.7	**0.74**	42	1.6
**8< = Hb<11 g/dl**	413	46.9	398	45.1		398	45.6		1,209	45.9
** Hb> = 11 g/dl**	455	51.7	470	53.2		459	52.6		1,384	52.5
** Mean (sd)**	11.03	1.3	10.98	1.2	**0.29**	11.02	1.3	**0.92**	11.01	1.2
** Median (interquartile range)**	11	1.6	11	1.5		11.1	1.6		11	1.5
**Birth weight** d										
** BW = >2.5 Kg**	776	89.3	779	90.3	**0.41**	745	87.3	**0.25**	2,300	89.0
** BW<2.5 Kg**	93	10.7	84	9.7		108	12.7		285	11.0
** Mean (sd)**	3	0.5	3.02	0.5	**0.43**	2.98	0.4	**0.06**	3	0.5
** Median (interquartile range)**	2.98	0.6	3	0.6		2.98	0.6		3	0.6
**Parasitaemia prevalence** e										
** Yes**	77	12.3	79	12.4	**0.59**	74	11.3	**0.97**	230	12.0
** No**	549	87.7	560	87.6		580	88.7		1,689	88.0
** GMPD**	79	8.4	86	8.6		80	7.1		245	8.0

**NOTE.** Data are the number and percentage of women assessed at 36 to 40 weeks of gestation (haemoglobin & parasitaemia) and at delivery (birth weight), unless otherwise indicated.

SP-IPTp: intermittent preventive treatment with SP.

IST-SP: intermittent screening and treatment with SP.

IST-AQAS: intermittent screening and treatment with amodiaquine plus artesunate combination.

GMPD; geometric mean of parasite density.

a Comparison was restricted to only women who remained within their randomization group, received two courses of SP-IPTp (IPT group) or were screened twice using an RDT at scheduled visits (IST groups) and in whom the primary outcome had been recorded.

b p<0.05 means observed differences between comparison groups is statistically significant or not significant if P>0.05.

c For this comparison of SP-IPT, SP-IST and AQAS-IST arms included 880, 883 and 872 women respectively.

d For this comparison of SP-IPT, SP-IST and AQAS-IST arms included 869, 863 and 853 women respectively.

e For this comparison of SP-IPT, SP-IST and AQAS-IST arms included 626, 639 and 654 women respectively.

**Figure 3:**
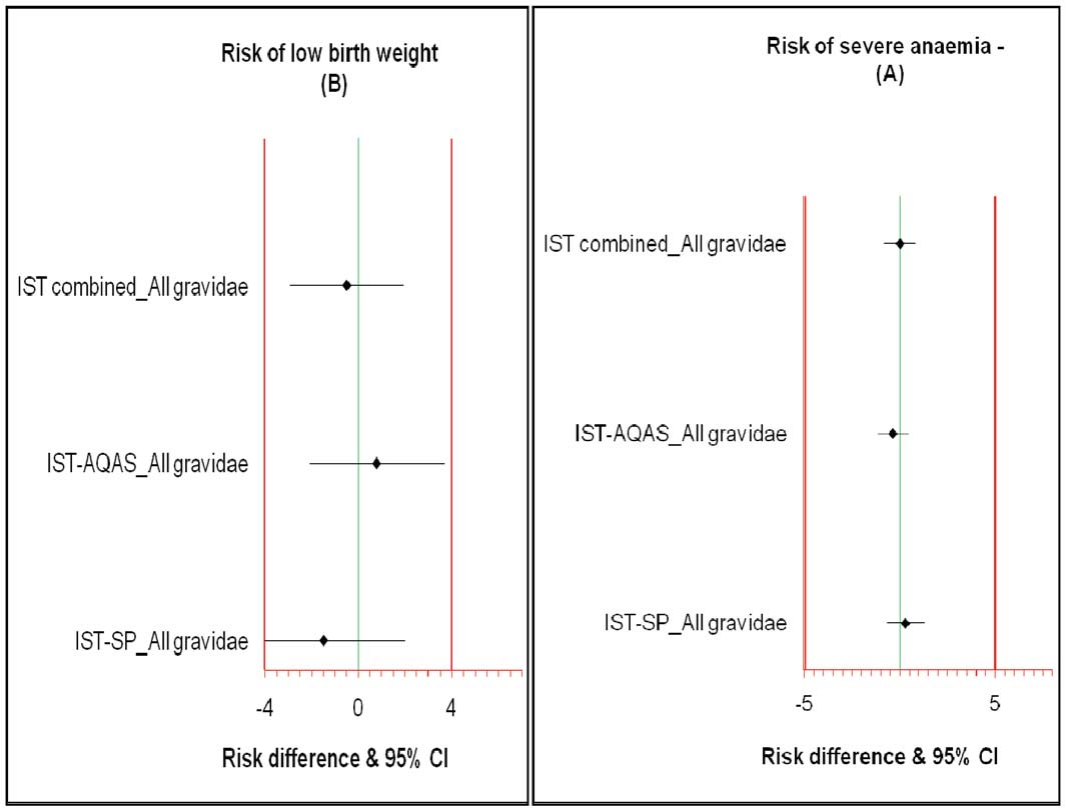
The effect of IST with AS + AS or SP compared with SP-IPTp on the risk of third trimester severe anaemia (panel A) or low birth weight (panel B) in women who received these interventions. The error bars shown indicate 2-sided 95% confidence intervals (CIs). The areas bound by −5 to 5 (panel 3A) and −4 to 4 (panel 3B) indicate zone of non-inferiority. In both panels the CIs lie to the left of the upper boundaries of the CIs and include zero indicating IST is non-inferior to SP-IPTp.

The risk of anaemia (severe or moderate) did not differ significantly between the three treatment groups across gravidity (Table 3). It was no higher in the IST groups than in women who received SP-IPTp; (RD = 0.29 [95% CI; -0.69-1.30] for IST-SP vs. SP-IPTp and RD = -0.36 [95% CI;-1.12-0.44] for IST-AQAS vs. SP-IPTp). The upper boundaries of the 2-sided 95% CI for the risk differences estimated between SP-IPTp and the IST groups were below the non-inferiority margin of 5% for third trimester severe anaemia, the main trial endpoint (Figure 3A). Third trimester severe anaemia, the primary trial end-point, was associated significantly in a univariate analysis with baseline parasitaemia baseline anaemia and young maternal age (p<0.05) (Table 3). In a multivariate analysis, which adjusted for the number of visits made by a woman, weeks of gestation at enrolment, gravidity and treatment group, third trimester severe anaemia remained significantly associated with baseline anaemia (p<0.0001) and young maternal age (p = 0.02) but only marginally with baseline parasitaemia (p = 0.07) (Table 3).

**Table 3:** Factors associated with third trimester severe anaemia in study women.

	3rd trimester severe anaemia			Unadjusted RR	(95%CI)	p-value^a^	Adjusted RR^b^	(95%CI)	p-value
	N	n	%						
**Treatment group**									
** SP-IPT**	880	12	1.36	1.00			1.00		
** IST-SP**	883	15	1.7	1.25	(0.59–2.65)	0.57	1.31	(0.63–2.75)	0.45
** AQAS-IST**	872	15	1.72	1.26	(0.59–2.68)	0.55	1.15	(0.54–2.44)	0.72
**Gravidity**									
** Multigravidae**	1,461	19	1.3	1.00			1.00		
** Secundigravidae**	590	9	1.53	1.17	(0.53–2.58)	0.69	0.49	(0.20–1.18)	0.11
** Primigravidae**	579	14	2.42	1.86	(0.94–3.68)	0.08	0.53	(0.23–1.24)	0.14
**Baseline parasitaemia**									
** No**	2,234	28	1.25	1.00			1.00		
** Yes**	398	14	3.52	2.81	(1.49 5.28)	0.001	1.90	(0.95–3.77)	0.07
**Baseline severe anaemia**									
** No**	2,545	30	1.18	1.00			1.00		
** Yes**	90	12	13.33	11.31	(5.99–21.36)	<0.001	7.74	(3.76–15.92)	<0.001
**Age category**									
** > = 30 years**	804	7	0.87	1.00			1.00		
** 25–29 years**	786	8	1.02	1.17	(0.42–3.21)	0.76	1.32	(0.48–3.66)	0.594
** < = 24 years**	993	26	2.62	3.01	(1.31–6.89)	0.01	3.29	(1.25–8.65)	0.02

RR: risk ratio; CI: confidence interval; IPT: intermittent preventive treatment; IST: intermittent screening and treatment.

a p<0.05 means observed differences between comparison groups is statistically significant or not significant if P>0.05.

b RRs were modeled using binomial regression. Treatment group, gravity, baseline parasitaemia and severe anaemia, and age category were included final model.

Similarly, the risk of low birth weight did not differ significantly between the three treatment groups regardless of gravidity; (RD = -1.17 [95% CI; -4.39-1.02] for IST-SP vs. SP-IPTp and RD = 0.78 [95% CI; -2.11-3.68] for IST-AQAS vs. SP-IPTp). The upper boundaries of the 2-sided 95% CI for the risk differences estimated between women in the SP-IPTp group and the IST lie below the non-inferiority margin of 4% set for low birth weight (Figure 3B). In a univariate analysis, low birth weight was associated significantly with baseline parasitaemia, low gravidity and young maternal age (p<0.05). However, in a multivariate analysis, which adjusted for the number of visits made by a woman, weeks of gestation at enrolment, gravidity and treatment group, only gravidity remained significantly associated with low birth weight. Primigravidae (RR = 2.10; 95%CI 1.49-2.95; p<0.0001) and secundigravidae (RR = 1.76; 95% CI 1.28-2.43; p = 0.001) were more likely to deliver low birth weight babies compared to multigravidae regardless of intervention arm. But the risk of low birth weight did not differ significantly between the three treatment groups across gravidity (Table 4).

**Table 4:** Factors associated with low birth weight of babies delivered by study women.

	Low birth weight			Unadjusted RR	(95%CI)	p-value^h^	Adjusted RR^i^	(95%CI)	p-value
	N	n	%						
**Treatment group**									
** SP-IPT**	868	92	10.6	1.00			1.00		
** IST-SP**	860	81	9.42	0.89	(0.67–1.18)	0.41	0.87	(0.65–1.15)	0.32
** AQAS-IST**	850	105	12.35	1.17	(0.89–1.52)	0.26	1.16	(0.89–1.51)	0.26
**Gravidity**									
** Multigravidae**	1,420	109	7.68	1.00			1.00		
** Secundigravidae**	581	75	12.91	1.68	(1.27–2.22)	<0.001	1.76	(1.28–2.43)	0.001
** Primigravidae**	572	94	16.43	2.14	(1.65–2.77)	<0.001	2.10	(1.49–2.95)	<0.001
**Baseline parasitaemia**									
** No**	2,175	219	10.07	1.00			1.00		
** Yes**	400	59	14.75	1.46	(1.12–1.91)	0.01	1.25	(0.94–1.65)	0.12
**Baseline severe anaemia**									
** No**	2,489	268	10.77	1.00			1.00		
** Yes**	89	10	11.24	1.04	(0.57–1.89)	0.89	0.82	(0.45–1.48)	0.51
**Age category**									
** > = 30 years**	771	67	8.69	1.00			1.00		
** 25–29 years**	996	115	11.55	1.07	(0.78–1.47)	0.68	0.86	(0.61–1.20)	0.37
** < = 24 years**	767	92	11.99	1.57	(1.19–2.07)	<0.001	0.93	(0.65–1.33)	0.7

**RR: risk ratio; CI: confidence interval; IPT: intermittent preventive treatment; IST: intermittent screening and treatment.**

h p<0.05 means observed differences between comparison groups is statistically significant or not significant if P>0.05.

i RRs were modeled using binomial regression. Treatment group, gravity, baseline parasitaemia and severe anaemia, and age category were included final model.

One hundred and forty-seven episodes of illness associated with malaria parasitaemia were recorded among study women between scheduled antenatal clinic visits; 44, 51 and 52 of these were in SP-IPTp, IST with SP, and IST with AS+ AQ groups respectively. The differences in the illness episodes are not statistically significant.

In all 2211 RDT tests were done in women in treatment groups 2 and 3 respectively of which 23% and 23% were positive and led to treatment. The risk of third trimester severe anaemia and low birth weight in those who were RDT negative throughout pregnancy and did not receive antimalarial treatment were no higher than in the SP-IPT arm. But the risk of parasitaemia at 36–40 weeks was higher in the SP-IPT arm than in the screening and treatment arms (Supplementary Table S1).

There were no statistically significant differences in the risk of preterm deliveries, abortions or perinatal births between the treatment groups (Supplementary Table S2). There were no maternal deaths. The proportion of women who reported adverse events during the 7 days following administration of SP-IPTp or following treatment with SP or AS+AQ did not differ significantly between the treatment groups with the exception of a complaint of general weakness (Supplementary Table S3) which was reported slightly more frequent in the group who received treatment with AS+AQ compared to the SP-IPT group (33.8% vs. 29.1%; p = 0.04).

### Study outcomes – intention to treat analysis

All primary and secondary outcomes were also analysed by intention to treat analysis and the results were very similar to those presented above (supplementary tables provided). Supplementary Table S4 show the comparison of key outcomes in women enrolled in SP-IPTp or IST groups while factors associated with third trimester severe anaemia and low birth weight are shown in supplementary Tables S5 and S6 respectively.

## Discussion

This study has shown that IST, using either SP or AS + AQ, was not inferior to IPTp with SP in preventing maternal anaemia and low birth weight, according to the non-inferiority criteria that were set prior to the trial, in women who used an LLIN in an area of moderately high malaria transmission in Ghana. There are a number of possible reasons why IST performed as well as SP-IPTp in our study. Firstly, it is possible that malaria is not an important cause of anaemia or low birth weight in the study area, in which case it would be difficult to detect an impact of different control strategies on the prevalence of these complications of malaria in pregnancy unless a much larger trial than the one we did was undertaken. We think that this is an unlikely explanation for our findings for several reasons. The study area is one of derived forest with perennial malaria transmission, although with a pronounced seasonal peak. The entomological inoculation rate in the neighbouring area of Kintampo was recently estimated to be 267 infective bites per year [17] and the prevalence of parasitaemia on presentation at the antenatal clinic among study women was 29.6% and 10.2% in primigravidae and in multigravidae respectively. These findings suggest that pregnant women in the study area are still being moderately exposed to malaria and that the level of transmission in the study area is comparable to that seen in communities where SP-IPT has been shown previously to have a significant impact [18], [19]. Furthermore we found an association between anaemia and malaria parasitaemia on presentation. A second possible explanation for our findings is that SP is no longer effective in the study area which would again make any comparisons between IST with SP and SP- IPTp unhelpful. In these circumstances, IST with AS+AQ would have been expected to show a better response than either IST or IPTp with SP and this was not the case. Furthermore, the adequate parasite clearance rate at day 28 in women who received a single dose of SP-IPTp was 85% (uncorrected) indicating only a moderate level of SP resistance. In an additional study carried out in the study area six months after completion of the study, the adequate parasite clearance rate by day 42 in 109 women was also 85% (unpublished data). Thus, it seems likely that IST with SP or AS+AQ can be as effective as SP-IPTp with the same drug in an area with moderate malaria transmission, marked seasonality and moderate SP resistance.

Demonstration of efficacy is only one aspect of the evaluation of a potentially new control tool; other important aspects are the ease with which it could be implemented, its acceptability and its cost. These aspects of IST have been evaluated in the current study and compared with those of SP-IPTp. The results of these studies will be presented in detail elsewhere. However, preliminary analysis of the findings studies indicates that IST can be introduced into a busy, antenatal clinic without disrupting its function. The approach appears acceptable to women who are used to receiving IPT with SP provided that the rationale for the intervention is explained to them [20].

Available evidence suggests that RDTs detect circulating parasite antigen and so compared to microscopy are better at detecting sub microscopic parasitaemia and may be a reliable indicator of placental infections [21]. An IST strategy draws on this and is heavily dependent upon the sensitivity and specificity of the RDT used to detect malaria infection. However, it is uncertain which kind of RDT would be most appropriate for diagnosing malaria in pregnancy. In general, assays based on HRP2 are considered more sensitive and more thermostable than those based on LDH and a sensitive test may be needed to detect parasites in the placenta. On the other hand HRP2 may persist in the circulation for several weeks after parasites have disappeared so that there is a danger that using an HRP2 test for repeated screening may result in women being treated unnecessarily when they attend for their next scheduled screening if they have been treated for an infection a month previously. Conversely a relatively insensitive test may not be able to detect an infection confined largely to the placenta. Few studies [21], [22], [23], [24] have reported good accuracy of RDTs used in the diagnoses of malaria in peripheral and placenta blood at delivery. However, studies of diagnostic accuracy in pregnant women attending antenatal clinics [25], [26], [27] are scarce and report a range of sentivities. In this study we found that the sensitivity and specificity of the OptiMAL test were 93.8% and 81% respectively compared to microscopy. A large study conducted by the WHO, with collaboration from FIND and CDC, has recently assessed the performance of about 40 commercially available RDTs in terms of their sensitivity, thermostability and ease of use [28]. This study is timely and will guide the choice of malaria RDTs in varying situations but more work is needed on defining the optimum test for detection of malaria in pregnancy.

No difference was found between the prevalence of peripheral blood parasitaemia between the treatment groups at 36–40 weeks of pregnancy or at six weeks post partum but the possibility that IST with SP or AS+AQ is not as effective as IPT with SP in preventing infection of the placenta cannot be excluded. This study had only limited financial resources which precluded analysis of the impact of each of the study interventions on the prevalence of placental malaria as measured by histology. How important such a difference might be in the absence of any apparent clinical adverse effects is uncertain but this needs to be investigated. Other weaknesses of this study are the absence of linked data on HIV positivity, although it is known that the overall prevalence of HIV positivity in the study population is relatively low, approximately 1.5% in the study clinics.

We are confident that the major finding of this study that IST is not inferior to SP-IPTp in the prevention of anaemia in pregnancy and low birth weight is sound and that IST is a potentially promising strategy for the control of malaria in pregnancy, especially in areas where the incidence of malaria is decreasing or where malaria is very seasonal. However, it is important that these encouraging findings including birth outcomes and maternal morbidity findings are confirmed in other geographical areas including parts of eastern and southern Africa, where SP is now failing since we have no alternative drug yet for SP and because the next generation of potential drugs are less likely to fulfill the same favourable profile of SP. As part of these it is also important that the impact of IST on placental malaria is investigated. A large multicentre trial of IST (clinicaltrials.gov identifier: NCT01084213) has started in four countries in Africa where malaria transmission is markedly seasonal will address these issues.

## Supporting Information

Checklist S1 (0.06 MB DOC)

Protocol S1 (0.16 MB DOC)

Table S1 Comparison of SP-IPTp with IST in women who were RDT negative throughout pregnancy and did not receive an antimalarial.
(0.06 MB DOC)

Table S2 Comparison of pregnancy outcomes for singleton births.
(0.03 MB DOC)

Table S3 Comparison of number of women who experienced adverse events within seven days of drug administration.
(0.04 MB DOC)

Table S4 Comparison of key outcomes in women enrolled in SP-IPTp or IST groups.
(0.06 MB DOC)

Table S5 Factors associated with third trimester severe anaemia in study women
(0.05 MB DOC)

Table S6 Factors associated with low birth weight of babies delivered by study women
(0.05 MB DOC)
