# Supplementary material for: Intermittent Screening and Treatment versus Intermittent Preventive Treatment of Malaria in Pregnancy: A Randomised Controlled Non-Inferiority Trial
Source: PLoS One. 2010 Dec 28;5(12):e14425. doi: 10.1371/journal.pone.0014425 (PMC3010999; doi:10.1371/journal.pone.0014425)
Supplement: Protocol S1 — (0.16 MB DOC) [file pone.0014425.s002.doc]

**RE-ENTRY GRANT PROPOSAL**

**TITLE OF PROPOSAL**

The effectiveness, cost and cost effectiveness of intermittent preventive treatment or screening and treatment of malaria in pregnancy among women using long lasting insecticide treated bed net: a randomised controlled trial.

**PRINCIPAL INVESTIGATOR**

Dr. Harry K. Tagbor School of Medical Sciences, KNUST, Kumasi, Ghana

**COLLABORATORS AND AFFILIATIONS**

Dr. Daniel Chandramohan London School of Hygiene and Tropical Medicine

Ms. Jane Bruce London School of Hygiene and Tropical Medicine

Dr. Edmund N.L Browne School of Medical Sciences, KNUST, Kumasi, Ghana

Prof. Brian Greenwood London School of Hygiene and Tropical Medicine

**PROPOSED STARTING DATE: November 1, 2006 DURATION: 24 Months**

**Abbreviations:**

AQ Adverse Event (s)

AS Acquired Immunodeficiency Syndrome

CRF(s) Case Record Form (s)

DSMB Data Safety Monitoring Board

EDT Early diagnosis and treatment of malaria

HIV Human Immunodeficiency Virus

IPT Intermittent Preventive Treatment

IPTp Intermittent Preventive Treatment in pregnancy

ITNs Insecticide treated mosquito net(s) or material (s)

LLIN Long lasting insecticide treated nets

LSHTM London School of Hygiene and Tropical Medicine

MCH Maternal and Child Health

OPD Outpatients Department

PCR Polymerase Chain Reaction

PI Principal Investigator

pLDH Plasmodium Lactate Dehydrogenase

SAE(s) Serious Adverse Event(s)

SP Sulphadoxine Pyrimethamine

TBA Traditional Birth Attendant

WBC White blood cell

WHO The World Health Organisation

**RESEARCH PROPOSAL**

# TITLE

The effectiveness, cost and cost effectiveness of intermittent preventive treatment or screening and treatment of malaria in pregnancy among women using long lasting insecticide treated bed net: a randomised controlled trial.

# BACKGROUND & RATIONALE

*Plasmodium falciparum* infection in pregnancy is associated with an increased risk of maternal and foetal complications including maternal anaemia and low birth weight (Brabin 1991; Menendez 1995). The pattern and severity of complications depend on gravidity, gestational age, age and HIV/AIDS and a woman’s pre-existing immunity, which is influenced by the local malaria transmission profile (WHO 2004). The WHO has recommended a package of interventions for preventing and controlling malaria infection in pregnancy in endemic areas which includes; the early diagnosis and treatment of malaria (EDT), intermittent preventive treatment during pregnancy (IPTp) using Sulphadoxine-Pyrimethamine (SP) and the use of insecticide treated bed nets (ITNs) (WHO 2004).

The policy of SP-IPTp for pregnant women has been shown to be efficacious, easy to administer, safe and tolerable (Pari*se et a*l. 1998; Verhoe*ff et a*l. 1998; Shulm*an et a*l. 1999; Rogers*on et a*l. 2000; van Ei*jk et a*l. 2004; Kayent*ao et a*l. 2005). Currently, SP-IPTp has been rated as having the most favourable effectiveness profile in terms of its low cost, compliance and reduced morbidity and mortality resulting from the prevention of maternal anaemia and low birth weight (Goodm*an et a*l. 2001; Newm*an et a*l. 2003). However, implementation of IPTp in most settings is limited by social, cultural, economic and operational challenges despite good coverage of antenatal services (Rogers*on et a*l. 2000; Hol*tz et a*l. 2004). In Ghana, implementation of SP-IPTp is currently being piloted in only 20 of its 110 districts (Dod*oo et a*l. 2005). The resistance to SP has been spreading across sub-Saharan Africa and thus the effectiveness of SP-IPTp has been questioned (Newm*an et a*l. 2003). There are very limited antimalarial drugs with plasma half life comparable to SP (e. g. Mefloquine) that could be used to replace SP for IPTp in areas with high SP resistance. Furthermore there is very limited data on the efficacy and safety during pregnancy of drugs with potentials to replace SP for IPTp. Thus evaluation of safety and efficacy of antimalarials drugs suitable for IPTp and alternative approaches to reduce the burden of malaria during pregnancy are needed urgently.

A recent study of rapid diagnostic test (RDT) screening and treatment of parasitaemic pregnant women with amodiaquine (AQ) or SP singly or their combination in Ghana showed that this approach can reduce the prevalence of low birth weight by 30% and the prevalence of severe maternal anaemia (Hb < 8.0g/dl) at delivery by 45% (Tagb*or et a*l. 2006). These protective effects are similar to those obtained with SP-IPTp in Mali (Kayent*ao et a*l. 2005); Kenya (Pari*se et a*l. 1998; Shulm*an et a*l. 1999; Nja*gi et a*l. 2003) and Malawi (Verhoe*ff et a*l. 1997; Rogers*on et a*l. 2000). Although RDTs are not quantitative (Wongsrichanalai 2001) and peripheral parasite density in pregnancy may not correlate with placental parasitaemia, the use of OptiMAL® dipsticks to detect circulating parasite-specific lactate dehydrogenase (pLDH) may be of public health significance. The antenatal RDT screening was feasible and the cost was comparable to that of microscopy which costs an average of 10,000 and 15,000 cedis (1 US$=9100 cedis) in public and mission hospitals respectively.

The results of initial studies of the effects of ITNs on maternal anaemia and low birth weight were inconsistent because of the complexity of factors that determine acquisition and continued use of the nets. For example studies in Ghana (Brow*ne et a*l. 2001) and Kenya (Shulm*an et a*l. 1998) did not find any effect on malaria and anaemia in pregnancy following the use of insecticide impregnated bed nets; whilst other studies in the mesoendemic area of the Thai-Burmese border (Dol*an et a*l. 1993) and in The Gambia (D'Alessand*ro et a*l. 1996) found reductions in maternal anaemia and low birth weight with the use of ITNs.

However, a recent study of the impact of large scale use of ITNs among Kenyan pregnant women (ter Kui*le et a*l. 2003; ter Kui*le et a*l. 2003) showed significant reductions in malaria related adverse pregnancy outcomes. It is not clear though, how much extra benefit there is in combining use of ITNs and IPT during pregnancy (Nja*gi et a*l. 2003; Mba*ye et a*l. 2006).

There is currently great international support and momentum to increase the coverage of ITNs use among pregnant women. Thus while looking for alternative drugs for IPT in areas with SP resistance we have to consider the additional benefits of the combination of ITN and IPT over ITN with effective case management approach and the potential drug related adverse outcomes of drugs used for IPTp. The RDT screening and treatment with an effective antimalarial of those with a positive RDT test will reduce the number of women that would be exposed to drugs. Although some women with placental malaria may be missed by the RDT screening, it can be argued that the proportion of women with placental malaria missed by RDT would be minimal if all women used an ITN and that the risk of missed placental malaria will be offset by the reduction in women exposed to antimalarials of uncertain safety profile unnecessarily.

We are proposing this follow up study to determine whether active case finding and management is as effective as intermittent preventative treatment for the control of malaria during pregnancy. A two-armed non-inferiority randomised trial using SP in both arms (LLIN+ IPTp or LLIN + RDT & treatment) is appropriate to address this question. However, if SP is not very effective, a difference may not be found between the two arms since the effect of SP as IPT or treatment would be minimal and the effect of LLIN would be similar in both arms. We cannot replace SP with another effective antimalarial in the IPTp arm since the safety profile of potential antimalarial drugs that could replace SP are not adequately established to be useful for IPTp. A two arm study of LLIN + SP-IPTp and LLIN + RDT & AQ+AS treatment will not be appropriate to prove the principle that LLIN + RDT & treatment is not inferior to LLIN + IPTp since AQ+AS is likely to be more efficacious than SP and this would bias our observation. Thus we propose a three arm trial: (1) LLIN plus SP-IPTp; (2) LLIN plus RDT and treatment with SP (3) LLIN plus RDT and treatment with AQ+AS.

AS+AQ is the first line drug of choice for treatment of uncomplicated malaria in Ghana. However there is very little safety data on the use of AS+AQ for the treatment of malaria during pregnancy and the safety data collected in this study will be very useful for informing the national drug policy for treatment of malaria during pregnancy.

# **METHODS**

## General objective

To show that the LLIN plus RDT screening and treatment strategy is not inferior to LLIN plus SP-IPTp in reducing the burden of malaria during pregnancy.

## Specific objectives

Primary

1. To demonstrate that the prevalence of severe anaemia (Hb < 8g/dl) at 34 to 36 weeks of gestation in the LLIN plus RDT screening and case management arm is not greater than that in the LLIN plus IPTp arm.

Secondary

1. To demonstrate that the prevalence of low birth weight (BW < 2500g) at delivery or within 72 hours after delivery in the LLIN plus RDT screening and case management arm is not greater than that in the LLIN plus IPTp arm.
2. To demonstrate that the prevalence of anaemia (Hb < 11g/dl) at 34 to 36 weeks of gestation in the LLIN plus RDT screening and case management arm is not greater than that in the LLIN plus IPTp arm.
3. To demonstrate that the prevalence of placenta parasitaemia at in the LLIN plus RDT screening and case management arm is not greater than that in the LLIN plus IPTp arm.
4. To demonstrate that the incidence of spontaneous abortions, intrauterine deaths/stillbirths, neonatal and maternal mortality and developmental delays in the LLIN plus RDT screening and case management arm is not greater than that in the LLIN plus IPTp arm.
5. To demonstrate that the cost per case of (1) severe maternal anaemia averted (2) maternal anaemia averted (3) peripheral malaria averted and (4) placenta malaria averted is not higher in the LLIN plus RDT screening and case management arm than in LLIN plus SP-IPTp arm.

## Study outcomes

Primary

- 1. Prevalence of severe maternal anaemia (Hb < 8g/dl) at 34 to 36 weeks of gestation.

Secondary

1. Prevalence of low birth weight (BW < 2500g) at delivery or within 72 hours of delivery.
2. Prevalence of maternal anaemia (Hb < 11g/dl) at 34 to 36 weeks of gestation.
3. Prevalence of placenta parasitaemia.
4. Incidence of post intervention malaria cases
5. Proportions of congenital anomalies in live births among the intervention groups stratified by gestation, gravidity, parity and age.
6. Proportions of spontaneous abortions, intrauterine death, stillbirths, neonatal and maternal mortality and pre-term deliveries.
7. Cost per severe maternal anaemia averted.
8. Cost per (non-severe) maternal anaemia averted.
9. Cost per peripheral malaria case averted.
10. Cost per placenta malaria averted.

## ***Sample size***

The prevalences of severe anaemia at delivery and low birth weight have been shown to be about 12% and 5.7% respectively among Gambian multigravidae who received varying doses of SP-IPT (Mba*ye et a*l. 2006). In the absence of local data from the proposed study site, we estimate that the prevalences of severe anaemia in the third trimester and low birth weight will be 12% and 6% respectively in the LLIN +SP-IPTp arm of this study, similar to the Gambian population.

For trial planning we have set a non-inferiority margin of 5%. This means we would deem the LLIN + RDT screening with SP or AQ+AS treatment options to be inferior to the LLIN +SP-IPTp option if the prevalence of severe anaemia at 34 to 36 weeks is 5% greater in the LLIN+RDT with SP or AS+AQ treatment arm than that is observed in the LLIN+SP-IPT arm. Since we expect the prevalence of severe anaemia in the LLIN +SP-IPTp arm would be 12%, we need 887 women per arm to detect a 5% difference between the two study arms at 95% significance with 90% power. Considering a 20% dropout rate we need 1110 women per arm.

Setting a non-inferiority margin of 5% seems clinically and statistically relevant; and yields a sample size that makes the execution of the study feasible within the time frame and with the resources available. Choosing a smaller margin than 5% may be too tight requiring large sample size of over 2000 per arm which might not be feasible and achievable within the time frame; a larger margin than 5% may be too liberal to compare the public health impact of these interventions.

We estimate that 925 women per arm can show with 90% power at 95% significance level and accounting for a 20% dropout rate, that the prevalence of low birth weight associated with the LLIN + RDT screening with or without SP or AQ+AS treatment is no worse than LLIN + SP-IPTp by a margin of 4%.

Since, a sample size of 1110 per arm is adequate to detect any effects of the interventions on both birth weight and maternal Hb, a total of 3330 pregnant women will be recruited into this study.

## Study Design

A three-arm open label randomised control non-inferiority trial of RDT screening with or without treatment with SP or AQ+AS versus IPTp using SP as follows: -

1. OptiMAL® antigen screening and treatment with SP plus LLIN
2. OptiMAL® antigen screening and treatment with AQ+AS plus LLIN
3. SP-IPTp plus LLIN

## Study site and population

The study will be conducted in the Ejisu-Juaben and Sekyere East districts of the Ashanti Region of Ghana. Enrolment will be done simultaneously at the antenatal clinics of the Effiduase and Juaben district hospitals and a sub district health centre in each of the districts. The Effiduase and Juaben hospitals have 50 and 40 beds respectively and provide all basic medical services including adult medicine, paediatrics, surgery and obstetrics and gynaecology. They run weekly antenatal clinic sessions and together they register an average of 220 new pregnant women per month. The sub district health centres (at Kwaso and Kumawu) are manned by qualified medical assistants and midwives. Together they register an average of 80 new pregnant women per month. All health facilities in the study districts including the proposed enrolling clinics offer SP-IPT to pregnant women following the MoH guidelines. Pregnant women in their second or third trimester diagnosed with malaria are treated with AQ+AS as recommended by the MoH at these facilities. The study population will comprise pregnant women presenting at the hospital’s antenatal clinic with a gestation of 16 to 24 weeks at their first booking.

## Enrolment Procedures

A schematic profile of the study is shown in Figure 1. All study pregnant women will be recruited from weekly antenatal clinic sessions held at the study sites over a period of one and half years. Three teams of midwives and their assistants put together and trained by the principal investigator (PI) will assist the PI and research teams to conduct the recruitment during antenatal clinic hours. The standard operating procedures at the clinic will be as follows:

1. Randomise pregnant women reporting at the ANC who consent to participate in the study into three arms: an IPTp arm, RDT screening and SP case management arm and RDT screening and AQ+AS case management arm. Distribute LLIN to eligible pregnant women in all arms.
2. Obtain blood for baseline investigations including haemoglobin level, thin and thick blood films for malaria parasite counts and, filter paper blood spots.
3. Assess all pregnant women in the RDT and treatment groups clinically and obstetrically prior to OptiMAL® dipstick screening; for those parasitaemic offer SP or AQ+AS and ingestion of first dose observed directly at the ANC. Encourage the women to regularly sleep under the bed nets provided and take their daily doses of iron and folic acid tablets.
4. For women in the RDT and treatment groups who are negative offer only daily doses of iron and folic acid tablets and encourage them to regularly sleep under the bed nets provided.
5. Women in the IPTp group will receive SP and ingestion directly observed at the ANC according to the national guideline. Encourage the women to regularly sleep under the bed nets provided and take their daily doses of iron and folic acid tablets.
6. Women who decline participation in the study will receive SP-IPTp according to the national guideline.

**Inclusion Criteria**

A pregnant woman will be eligible to be included in the study if: -

1. Her pregnancy is confirmed at 16 to 24 weeks at their first booking.
2. She is willing to participate and complete the test schedule, and has given informed consent.
3. She is willing to have supervised delivered at maternity units in the district.
4. She lives within the study district.

**Exclusion Criteria**

A pregnant woman will not be eligible for inclusion in the study if: -

1. She has a past obstetric and medical history that will adversely affect the interpretation of outcomes such as repeated stillbirths and eclampsia.
2. She has a haemoglobin level below 5.0 g/dl.
3. She has malaria that is severe enough to require parenteral medication.

## Antenatal Follow-Up Procedures

1. **Day 7:** Women receiving SP or AQ+AS treatment will be followed up actively on the day 7 post treatment to assess any adverse events during the week post treatment.
2. **At 6 months:** repeat SP-IPT for women in the IPT arm and treat women in the case management with SP or AQ+AS if they are RDT positive. Women in the case management arm with negative RDT would receive only haematinics. Ask all study women to regularly sleep under bed nets provided.
3. **At 8 months:** repeat SP-IPT for women in the IPT arm and treat women in the case management with SP or AQ+AS if they are RDT positive. Women in the case management arm with negative RDT would receive only haematinics. Ask all study women to regularly sleep under bed nets provided.
4. **At 34 to 36 weeks:** actively follow up women to sample blood by finger prick for haemoglobin measurements, slide smears for malaria parasite counts and filter paper blood spots.
5. **Unscheduled visits:** Women presenting with a history of fever during an unscheduled visit will be tested for malaria parasite. If they were found parasitaemic, they will be deemed to be treatment failure and will be treated with quinine as rescue medication.
6. All pregnant women would receive haematinics packs at each of the follow ups.

## Delivery and Postpartum Follow-up

1. The midwives will record all birth weights and note any stillbirths and perinatal deaths.
2. Midwives will record all congenital deformities for verification and confirmation by a clinician.
3. Visit all women and babies at 6 to 8 weeks post delivery to obtain reports of any neonatal adverse events such as deaths or morbidity.
4. At 6 weeks post partum, if a woman has peripheral parasitaemia she will be treated with SP and followed up for parasite clearance on days 14 and 28 post treatment. If there is no parasite clearance on day 14 and/or day 28 a course of rescue medication (quinine) will be given. This part of the study is necessary to assess the efficacy of SP to clear parasitaemia.

Figure 1: - Flow chart of proposed study design

**RDT screening and SP case management arm**

Record pregnancy and delivery outcomes (abortions, SB, abnormalities); BW and neonatal jaundice.

Follow-up child at 6 to 8 weeks

Check blood slide of the mother for MP. If positive give a course of SP and follow up on day 14 and day 28

**LLIN plus SP-IPTp and add daily doses of haematinics.**

**LLIN and treatment with SP and daily doses of haematinics for RDT positive cases.**

Randomise pregnant women of 16 – 24 weeks gestation into IPTp and case finding and management arms at ANC sessions.

Passive follow up at 6, 8 & 9 months. Repeat SP-IPTp. Hb at 34 to 36 weeks

**IPTp Arm (not screened)**

**LLIN and daily doses of haematinics only for RDT negative women**

Passive follow up at 6, 8 & 9 months. Repeat RDT screening and treat with SP if she is parasitaemic. Hb at 34 to 36 weeks.

Record pregnancy and delivery outcomes (abortions, SB, abnormalities); BW and neonatal jaundice.

Follow-up child at 6 to 8 weeks

Check blood slide of the mother for MP. If positive give a course of SP and follow up on day 14 and day 28

**RDT screening and AQ+AS case management arm**

**LLIN and treatment with AQ+AS and daily doses of haematinics for RDT positive cases.**

**LLIN and daily doses of haematinics only for RDT negative women**

Passive follow up at 6, 8 & 9 months. Repeat RDT screening and treat with SP if she is parasitaemic. Hb at 34 to 36 weeks.

Record pregnancy and delivery outcomes (abortions, SB, abnormalities); BW and neonatal jaundice.

Follow-up child at 6 to 8 weeks

Check blood slide of the mother for MP. If positive give a course of SP and follow up on day 14 and day 28

# **REFERENCES**

Brabin B. J. (1991). The risks and severity of malaria in pregnant women. Geneva, World Health Organization**:** 1-34.

Browne E. N. L., Maude G. H. and Binka F. N. (2001). "The impact of insecticide-treated bednets on malaria and anaemia in pregnancy in Kassena-Nankana district, Ghana: a randomized controlled trial." Tropical Medicine and International Health **6**: 667-676.

D'Alessandro U., Langerock P., Bennett S., Francis N., Cham K. and Greenwood B. M. (1996). "The impact of a national impregnated bed net programme on the outcome of pregnancy in primigravidae in The Gambia." Transactions of the Royal Society of Tropical Medicine & Hygiene **90**: 487-492.

Dodoo A. N. O., Gyansa-Lutterodt M., Frempong N., Thompson H., Amofa G., Bart-Plange C., Allotey N. K. and Eghan K. E. (2005). "Preliminary safety assessment of sulphadoxine-pyrimethamine during intermittent presumptive treatment of pregnant women in a region with high prevalence of G6PD deficiency." International Journal of Risk & Safety in Medicine

**17**: 13-18.

Dolan G., ter Kuile F. O., Jacoutot V., White N. J., Luxemburger C., Malankirii L., Chongsuphajaisiddhi T. and Nosten F. (1993). "Bed nets for the prevention of malaria and anaemia in pregnancy." Transactions of the Royal Society of Tropical Medicine & Hygiene **87**: 620-626.

Goodman C. A., Coleman P. G. and Mills A. J. (2001). "The cost-effectiveness of antenatal malaria prevention in sub-Saharan Africa." Am J Trop Med Hyg **64**: 45-56.

Holtz T. H., Kachur S. P., Roberts J. M., Marum L. H., Mkandala C., Chizani N., Macheso A. and Parise M. E. (2004). "Use of antenatal care services and intermittent preventive treatment for malaria among pregnant women in Blantyre District, Malawi." Tropical Medicine & International Health **9**: 77-82.

Kayentao K., Kodio M., Newman R. D., Maiga H., Doumtabe D., Ongoiba A., Coulibaly D., Keita A. S., Maiga B., Mungai M., Parise M. E. and Doumbo O. (2005). "Comparison of intermittent preventive treatment with chemoprophylaxis for the prevention of malaria during pregnancy in Mali." J Infect Dis **191**: 109-116.

Mbaye A., Richardson K., Balajo B., Dunyo S., Shulman C., Milligan P., Greenwood B. and Walraven G. (2006). "A randomised, placebo- controlled trial of intermittent preventative treatment with sulphadoxine-pyrimethamine in Gambian multigravidae." Tropical Medicine and International Health **11**(In press).

Menendez C. (1995). "Malaria during pregnancy: a priority area of malaria research and control." Parasitology Today **11**: 178-183.

Newman R. D., Parise M. E., Slutsker L., Nahlen B. and Steketee R. W. (2003). "Safety, efficacy and determinants of effectiveness of antimalarial drugs during pregnancy: Implications for prevention programmes in Plasmodium falciparum-endemic sub-Saharan Africa." Tropical Medicine & International Health **8**: 488-506.

Njagi J. K., Magnussen P., Estambale B., Ouma J. and Mugo B. (2003). "Prevention of anaemia in pregnancy using insecticide-treated bednets and sulfadoxine-pyrimethamine in a highly malarious area of Kenya: a randomized controlled trial." Trans R Soc Trop Med Hyg **97**: 277-282.

Parise M. E., Ayisi J. G., Nahlen B. L., Schultz L. J., Roberts J. M., Misore A., Muga R., Oloo A. J. and Steketee R. W. (1998). "Efficacy of sulfadoxine-pyrimethamine for prevention of placental malaria in an area of Kenya with a high prevalence of malaria and human immunodeficiency virus infection." Am J Trop Med Hyg **59**: 813-822.

Rogerson S. J., Chaluluka E., Kanjala M., Mkundika P., Mhango C. and Molyneux M. E. (2000). "Intermittent sulfadoxine-pyrimethamine in pregnancy: effectiveness against malaria morbidity in Blantyre, Malawi, in 1997-99." Transactions of the Royal Society of Tropical Medicine & Hygiene **94**: 549-553.

Shulman C. E., Dorman E. K., Cutts F., Kawuondo K., Bulmer J. N., Peshu N. and Marsh K. (1999). "Intermittent sulphadoxine-pyrimethamine to prevent severe anaemia secondary to malaria in pregnancy: a randomised placebo-controlled trial." Lancet **353**: 632-636.

Shulman C. E., Dorman E. K., Talisuna A. O., Lowe B. S., Nevill C., Snow R. W., Jilo H., Peshu N., Bulmer J. N., Graham S. and Marsh K. (1998). "A community randomized controlled trial of insecticide-treated bednets for the prevention of malaria and anaemia among primigravid women on the Kenyan coast." Tropical Medicine & International Health **3**: 197-204.

Tagbor H., Bruce J., Browne E., Randall A., Greenwood B. and Chandramohan D. (2006). "Efficacy, safety and tolerability of amodiaquine and sulphadoxine-pyrimethamine used singly or in combination for the treatment of malaria in pregnancy." (Submitted).

ter Kuile F. O., Terlouw D. J., Kariuki S. K., Phillips-Howard P. A., Mirel L. B., Hawley W. A., Friedman J. F., Shi Y. P., Kolczak M. S., Lal A. A., Vulule J. M. and Nahlen B. L. (2003). "Impact of permethrin-treated bed nets on malaria, anemia, and growth in infants in an area of intense perennial malaria transmission in western Kenya." Am J Trop Med Hyg **68**: 68-77.

ter Kuile F. O., Terlouw D. J., Phillips-Howard P. A., Hawley W. A., Friedman J. F., Kariuki S. K., Shi Y. P., Kolczak M. S., Lal A. A., Vulule J. M. and Nahlen B. L. (2003). "Reduction of malaria during pregnancy by permethrin-treated bed nets in an area of intense perennial malaria transmission in western Kenya." Am J Trop Med Hyg **68**: 50-60.

van Eijk A. M., Ayisi J. G., ter Kuile F. O., Otieno J. A., Misore A. O., Odondi J. O., Rosen D. H., Kager P. A., Steketee R. W. and Nahlen B. L. (2004). "Effectiveness of intermittent preventive treatment with sulphadoxine-pyrimethamine for control of malaria in pregnancy in western Kenya: a hospital-based study." Trop Med Int Health **9**: 351-360.

Verhoeff F. H., Brabin B. J., Chimsuku L., Kazembe P., Russell W. B. and Broadhead R. L. (1998). "An evaluation of the effects of intermittent sulfadoxine-pyrimethamine treatment in pregnancy on parasite clearance and risk of low birthweight in rural Malawi." Annals of Tropical Medicine & Parasitology **92**: 141-150.

Verhoeff F. H., Brabin B. J., Masache P., Kachale B., Kazembe P. and Van der Kaay H. J. (1997). "Parasitological and haematological responses to treatment of Plasmodium falciparum malaria with sulphadoxine-pyrimethamine in southern Malawi." Ann Trop Med Parasitol **91**: 133-140.

WHO (2004). A Strategic Framework for Malaria Prevention and Control during Pregnancy in the African Region. Brazzaville, WHO Regional Office for Africa**:** AFR/MAL/04/01.

Wongsrichanalai C. (2001). "Rapid diagnostic techniques for malaria control." Trends in Parasitology **17**: 307-309.
