# Supplementary material for: Intermittent Screening and Treatment versus Intermittent Preventive Treatment of Malaria in Pregnancy: A Randomised Controlled Non-Inferiority Trial
Source: PLoS One. 2010 Dec 28;5(12):e14425. doi: 10.1371/journal.pone.0014425 (PMC3010999; doi:10.1371/journal.pone.0014425)
Supplement: Table S1 — Comparison of SP-IPTp with IST in women who were RDT negative throughout pregnancy and did not receive an antimalarial. (0.06 MB DOC) [file pone.0014425.s003.doc]

Table S1: - Comparison[[1]](#footnote-2) of SP-IPTp with IST in women who were RDT negative throughout pregnancy and did not receive an antimalarial.

|  | **SP-IPTp** |  | **IST-SP** |  |  | **IST-AQAS** |  |  | Total |  |
| --- | --- | --- | --- | --- | --- | --- | --- | --- | --- | --- |
|  | **n** | **%** | **n** | **%** | ***p-value[[2]](#footnote-3)*** | **n** | **%** | ***p-value*** | **n** | **%** |
| **Haemoglobin** |  |  |  |  |  |  |  |  |  |  |
| **Hb<8g/dl** | 12 | 1.4 | 9 | 1.3 | ***0.75*** | 8 | 1.2 | ***0.65*** | 29 | 1.3 |
| **8<=Hb<11g/dl** | 413 | 46.9 | 308 | 45.0 |  | 296 | 44.7 |  | 1,017 | 45.7 |
| **Hb>=11g/dl** | 455 | 51.7 | 367 | 53.7 |  | 358 | 54.1 |  | 1,180 | 53.0 |
| **Mean (sd)** | 11.03 | 1.3 | 10.98 | 1.2 |  | 11.02 | 1.3 |  | 11.01 | 1.2 |
| **Median (interquartile range)** | 11 | 1.6 | 11 | 1.5 |  | 11.1 | 1.6 |  | 11 | 1.5 |
|  |  |  |  |  |  |  |  |  |  |  |
| **Birth weight** |  |  |  |  |  |  |  |  |  |  |
| **BW=>2.5Kg** | 776 | 89.3 | 779 | 90.3 | ***0.62*** | 745 | 87.3 | ***0.70*** | 2,300 | 89.0 |
| **BW<2.5Kg** | 93 | 10.7 | 84 | 9.7 |  | 108 | 12.7 |  | 285 | 11.0 |
| **Mean (sd)** | 3 | 0.5 | 3.02 | 0.5 |  | 2.98 | 0.4 |  | 3 | 0.5 |
| **Median (interquartile range)** | 2.98 | 0.6 | 3 | 0.6 |  | 2.98 | 0.6 |  | 3 | 0.6 |
|  |  |  |  |  |  |  |  |  |  |  |
| **Parasitaemia prevalence** |  |  |  |  |  |  |  |  |  |  |
| **Yes** | 77 | 12.3 | 79 | 12.4 | ***0.01*** | 74 | 11.3 | ***0.00*** | 230 | 12.0 |
| **No** | 549 | 87.7 | 560 | 87.6 |  | 580 | 88.7 |  | 1,689 | 88.0 |
| **GMPD** | 79 | 8.4 | 41 | 2.8 |  | 39 | 2.6 |  | 159 | 4.7 |
|  |  |  |  |  |  |  |  |  |  |  |

**NOTE.** Data are the number and percentage of women assessed at 36 to 40 weeks of gestation (haemoglobin & parasitaemia) and at delivery (birth weight), unless otherwise indicated.

SP-IPTp: intermittent preventive treatment with SP

IST-SP: intermittent screening and treatment with SP

IST-AQAS: intermittent screening and treatment with amodiaquine plus artesunate combination

GMPD; geometric mean of parasite density

1. Comparison is restricted to only women who tested RDT negative throughout the study period and did not receive an antimalarial. [↑](#footnote-ref-2)
2. p<0.05 means observed differences between comparison groups is statistically significant or not significant if P>0.05 [↑](#footnote-ref-3)
