# Supplementary material for: Intermittent Screening and Treatment versus Intermittent Preventive Treatment of Malaria in Pregnancy: A Randomised Controlled Non-Inferiority Trial
Source: PLoS One. 2010 Dec 28;5(12):e14425. doi: 10.1371/journal.pone.0014425 (PMC3010999; doi:10.1371/journal.pone.0014425)
Supplement: Table S2 — Comparison of pregnancy outcomes for singleton births. (0.03 MB DOC) [file pone.0014425.s004.doc]

Table S2: - Comparison of delivery outcomes for singleton births.

|  | **SP-IPTp** | | **IST-SP** | |  | **IST-AQAS** | |  | **Total** | |
| --- | --- | --- | --- | --- | --- | --- | --- | --- | --- | --- |
|  | **n** | **%** | **n** | **%** | **p-value** | **n** | **%** | **p-value** | **n** | **%** |
| **Term deliveries** | 875 | 95.4 | 853 | 95.5 |  | 859 | 95.9 |  | 2587 | 95.6 |
| **Preterm deliveries** | 15 | 1.6 | 19 | 2.1 | **0.4** | 15 | 1.7 | **0.9** | 49 | 1.8 |
| **Abortions** | 12 | 1.3 | 5 | 0.6 | **0.1** | 6 | 0.7 | **0.2** | 23 | 0.9 |
| **Perinatal deaths** | 15 | 1.6 | 16 | 1.8 | **0.8** | 16 | 1.8 | **0.8** | 47 | 1.7 |
|  |  |  |  |  |  |  |  |  |  |  |
