# Supplementary material for: Intermittent Screening and Treatment versus Intermittent Preventive Treatment of Malaria in Pregnancy: A Randomised Controlled Non-Inferiority Trial
Source: PLoS One. 2010 Dec 28;5(12):e14425. doi: 10.1371/journal.pone.0014425 (PMC3010999; doi:10.1371/journal.pone.0014425)
Supplement: Table S3 — Comparison of number of women who experienced adverse events within seven days of drug administration. (0.04 MB DOC) [file pone.0014425.s005.doc]

Table S3: - Comparison of number of women who experienced adverse events within seven days of drug administration.

|  | **SP-IPTp** | | **IST-SP** | |  | **IST-AQAS** | |  | **Total** | |
| --- | --- | --- | --- | --- | --- | --- | --- | --- | --- | --- |
|  | **n** | **%** | **n** | **%** | **p-value** | **n** | **%** | **p-value** | **n** | **%** |
| **Any** | 388 | 42.3 | 379 | 42.4 | **0.9** | 405 | 45.2 | **0.2** | 1,172 | 43.3 |
| **General weakness** | 216 | 23.6 | 210 | 23.5 | **0.9** | 250 | 27.9 | **0.03** | 676 | 25.0 |
| **Headache** | 170 | 18.5 | 188 | 21.1 | **0.1** | 185 | 20.7 | **0.3** | 543 | 20.1 |
| **Vomiting** | 126 | 13.7 | 121 | 13.6 | **0.9** | 146 | 16.3 | **0.1** | 393 | 14.5 |
| **Nausea** | 111 | 12.1 | 122 | 13.7 | **0.4** | 128 | 14.3 | **0.9** | 361 | 13.3 |
| **Dizziness** | 97 | 10.6 | 103 | 11.5 | **0.5** | 100 | 11.2 | **0.7** | 300 | 11.1 |
| **Routine activities inhibited** | 80 | 8.7 | 84 | 9.4 | **0.3** | 94 | 10.5 | **0.2** | 258 | 9.5 |
| **Itching** | 67 | 7.3 | 56 | 6.3 | **0.6** | 66 | 7.4 | **0.2** | 189 | 7.0 |
|  |  |  |  |  |  |  |  |  |  |  |
