# Supplementary material for: Intermittent Screening and Treatment versus Intermittent Preventive Treatment of Malaria in Pregnancy: A Randomised Controlled Non-Inferiority Trial
Source: PLoS One. 2010 Dec 28;5(12):e14425. doi: 10.1371/journal.pone.0014425 (PMC3010999; doi:10.1371/journal.pone.0014425)
Supplement: Table S4 — Comparison of key outcomes in women enrolled in SP-IPTp or IST groups. (0.06 MB DOC) [file pone.0014425.s006.doc]

Table S4: Comparison[[1]](#footnote-2) of key outcomes in women enrolled in SP-IPTp or IST groups.

|  | **SP-IPTp** |  | **IST-SP** |  |  | **IST-AQAS** |  |  | Total |  |
| --- | --- | --- | --- | --- | --- | --- | --- | --- | --- | --- |
|  | **n** | **%** | **n** | **%** | **p-value** | **n** | **%** | **p-value** | **n** | **%** |
| **Haemoglobin** |  |  |  |  |  |  |  |  |  |  |
| **Hb<8g/dl** | 12 | 1.35 | 15 | 1.67 |  | 16 | 1.8 |  | 43 | 1.61 |
| **8<=Hb<11g/dl** | 417 | 47.07 | 401 | 44.65 |  | 409 | 45.96 |  | 1,227 | 45.89 |
| **Hb>=11g/dl** | 457 | 51.58 | 482 | 53.67 |  | 465 | 52.25 |  | 1,404 | 52.51 |
| **Mean (sd)** | 11.03 | 1.25 | 10.99 | 1.21 |  | 11.01 | 1.25 |  | 11.01 | 1.24 |
| **Median (interquartile range)** | 11 | 1.6 | 11 | 1.5 |  | 11 | 1.6 |  | 11 | 1.5 |
|  |  |  |  |  |  |  |  |  |  |  |
| **Birth weight (kg)** |  |  |  |  |  |  |  |  |  |  |
| **BW=>2.5Kg** | 796 | 88.64 | 794 | 89.21 |  | 767 | 86.47 |  | 2,357 | 88.11 |
| **BW<2.5Kg** | 102 | 11.36 | 96 | 10.79 |  | 120 | 13.53 |  | 318 | 11.89 |
| **Mean (sd)** | 2.99 | 0.48 | 3 | 0.48 |  | 2.96 | 0.46 |  | 2.98 | 0.47 |
| **Median (interquartile range)** | 2.96 | 0.59 | 3 | 0.6 |  | 2.96 | 0.59 |  | 2.99 | 0.57 |
|  |  |  |  |  |  |  |  |  |  |  |
| **Parasitaemia prevalence** |  |  |  |  |  |  |  |  |  |  |
| **Yes** | 558 | 87.87 | 581 | 87.63 |  | 597 | 88.97 |  | 1,736 | 88.17 |
| **No** | 77 | 12.13 | 82 | 12.37 |  | 74 | 11.03 |  | 233 | 11.83 |
| **GMPD** | 80 | 8.32 | 89 | 8.62 |  | 81 | 7.01 |  | 250 | 7.97 |
|  |  |  |  |  |  |  |  |  |  |  |

**NOTE.** Data are the number and percentage of women assessed at 36 to 40 weeks of gestation (haemoglobin & parasitaemia) and at delivery (birth weight), unless otherwise indicated.

1. In this comparison, women were included if they had received an initial treatment of IPTp or had had an initial screening test done and provided that an outcome had been recorded. [↑](#footnote-ref-2)
