# Supplementary material for: Intermittent Screening and Treatment versus Intermittent Preventive Treatment of Malaria in Pregnancy: A Randomised Controlled Non-Inferiority Trial
Source: PLoS One. 2010 Dec 28;5(12):e14425. doi: 10.1371/journal.pone.0014425 (PMC3010999; doi:10.1371/journal.pone.0014425)
Supplement: Table S5 — Factors associated with third trimester severe anaemia in study women (0.05 MB DOC) [file pone.0014425.s007.doc]

Table S5: Factors associated with third trimester severe anaemia in study women

|  | **3rd trimester severe anaemia** | | | **Unadjusted RR[[1]](#footnote-2)** | **(95%CI)** | **p-value** | **Adjusted RR** | **(95%CI)** | **p-value (LR test)** |
| --- | --- | --- | --- | --- | --- | --- | --- | --- | --- |
|  | **N** | **n** | **%** |  |  |  |  |  |  |
| **Treatment group** |  |  |  |  |  |  |  |  |  |
| **SP-IPT** | 886 | 12 | 1.35 |  |  |  |  |  |  |
| **IST-SP** | 898 | 15 | 1.67 | 1.23 | (0.58 - 2.62) | 0.59 | 1.24 | (0.59 - 2.61) | 0.56 |
| **AQAS-IST** | 890 | 16 | 1.8 | 1.33 | (0.63 - 2.57) | 0.46 | 1.11 | (0.53 - 2.34) | 0.78 |
| **Gravidity** |  |  |  |  |  |  |  |  |  |
| **Multigravidae** | 1,479 | 19 | 1.28 |  |  |  |  |  |  |
| **Secundigravidae** | 598 | 9 | 1.51 | 1.17 | (0.53 - 2.57) | 0.69 | 0.50 | (0.21 - 1.22) | 0.13 |
| **Primigravidae** | 592 | 15 | 2.53 | 1.97 | (1.01 - 3.86) | 0.05 | 0.71 | (0.31 - 1.59) | 0.41 |
| **Baseline parasitaemia** |  |  |  |  |  |  |  |  |  |
| **No** | 2,264 | 29 | 1.28 |  |  |  |  |  |  |
| **Yes** | 407 | 14 | 3.44 | 2.69 | (1.43 - 5.04) | 0.002 | 1.88 | (0.94 - 3.73) | 0.07 |
| **Baseline severe anaemia** |  |  |  |  |  |  |  |  |  |
| **No** | 2,581 | 31 | 1.2 |  |  |  |  |  |  |
| **Yes** | 93 | 12 | 12.9 | 10.74 | (5.70 - 20.24) | 0 | 7.58 | (3.66 - 15.70) | 0 |
| **Age category** |  |  |  |  |  |  |  |  |  |
| **>=30 years** | 813 | 7 | 0.86 |  |  |  |  |  |  |
| **25-29 years** | 1,014 | 13 | 1.28 | 1.49 | (0.60 - 3.71) | 0.39 | 1.46 | (0.56 - 3.80) | 0.44 |
| **<=24 years** | 795 | 22 | 2.77 | 3.21 | (1.38 - 7.48) | 0.01 | 3.27 | (1.25 - 8.54) | 0.02 |

RR: risk ratio; CI: confidence interval; IPT: intermittent preventive treatment; IST: intermittent screening and treatment

1. ?RRs were modeled using binomial regression. Treatment group, gravity, baseline parasitaemia and severe anaemia, and age category were included final model. [↑](#footnote-ref-2)
