# Supplementary material for: Intermittent Screening and Treatment versus Intermittent Preventive Treatment of Malaria in Pregnancy: A Randomised Controlled Non-Inferiority Trial
Source: PLoS One. 2010 Dec 28;5(12):e14425. doi: 10.1371/journal.pone.0014425 (PMC3010999; doi:10.1371/journal.pone.0014425)
Supplement: Table S6 — Factors associated with low birth weight of babies delivered by study women (0.05 MB DOC) [file pone.0014425.s008.doc]

Table S6: Factors associated with low birth weight of babies delivered by study women

|  | **Low birth weight** | | | **Unadjusted RR[[1]](#footnote-2)** | **(95%CI)** | **p-value** | **Adjusted RR** | **(95%CI)** | **p-value** |
| --- | --- | --- | --- | --- | --- | --- | --- | --- | --- |
|  | **N** | **n** | **%** |  |
| **Treatment group** |  |  |  |  |  |  |  |  |  |
| **SP-IPT** | 898 | 102 | 11.36 |  |  |  |  |  |  |
| **IST-SP** | 890 | 96 | 10.79 | 0.95 | (0.73 - 1.24) | 0.7 | 0.92 | (0.71 - 1.20) | 0.55 |
| **AQAS-IST** | 887 | 120 | 13.53 | 1.19 | (0.93 - 1.52) | 0.17 | 1.20 | (0.94 - 1.53) | 0.15 |
| **Gravidity** |  |  |  |  |  |  |  |  |  |
| **Multigravidae** | 1,457 | 124 | 8.51 |  |  |  |  |  |  |
| **Secundigravidae** | 605 | 84 | 13.88 | 1.63 | (1.26 - 2.12) | 0 | 1.74 | (1.29 - 2.34) | 0 |
| **Primigravidae** | 608 | 110 | 18.09 | 2.13 | (1.67 - 2.70) | 0 | 2.15 | (1.61 - 2.88) | 0 |
| **Baseline parasitaemia** |  |  |  |  |  |  |  |  |  |
| **No** | 2,249 | 250 | 11.12 |  |  |  |  |  |  |
| **Yes** | 423 | 68 | 16.08 | 1.45 | (1.13 - 1.85) | 0.003 | 1.24 | (0.96 - 1.60) | 0.10 |
| **Baseline severe anaemia** |  |  |  |  |  |  |  |  |  |
| **No** | 2,582 | 307 | 11.89 |  |  |  |  |  |  |
| **Yes** | 93 | 11 | 11.83 | 0.99 | (0.57 - 1.75) | 0.99 | 0.79 | (0.45 - 1.39) | 0.42 |
| **Age category** |  |  |  |  |  |  |  |  |  |
| **>=30 years** | 790 | 74 | 9.37 |  |  |  |  |  |  |
| **25-29 years** | 1,037 | 131 | 12.63 | 1.35 | (1.03 - 1.77) | 0.03 | 0.94 | (0.70 - 1.28) | 0.71 |
| **<=24 years** | 802 | 108 | 13.47 | 1.44 | (1.09 - 1.90) | 0.01 | 0.90 | (0.64 - 1.25) | 0.53 |

RR: risk ratio; CI: confidence interval; IPT: intermittent preventive treatment; IST: intermittent screening and treatment

1. ?RRs were modeled using binomial regression. Treatment group, gravity, baseline parasitaemia and severe anaemia, and age category were included final model. [↑](#footnote-ref-2)
